# Relative Values of Principles of Extension, Suspension and Mobility

**Published:** 1919-01

**Authors:** 


**The Relative Values of the Principles of Extension, Suspension, and Mobility. The Hodgen Wire Cradle Extension Suspension Splint.** By F. G. Nifong, M. D. *Journal of the American Medical Association*, Sept. 21, 1918.

The author states that the principles upon which rational fracture treatment is based have been carefully studied. These principles should be applied to the femur, the latter being the longest bone with the heaviest musculature, but they are identical for the humerus and other bones of the extremities.

*Principle of Fixation.* The value of fixation is unquestioned, as the site of fracture must be immobilized. In many cases, however, the importance given to fixation has prevented the application of other principles equally necessary for complete functional recovery, such as extension, especially in fractures of the long bones. Extension is essential chiefly in the case of fractured femurs, in order to prevent final deformity and loss of function. The application of extension, independently of the methods used, is really made through stretching the great fascia lata, which makes all soft parts and the bone resume their proper position.

Extension and the accompanying counter-extension should be guided by a knowledge of mechanics, and muscle attachment, and by X-ray examinations, so as to avoid shortening and displacement.

*Suspension and Mobility.* Mobility of the patient is an important factor increasing the chances of recovery. It is obtained in suspended splints allowing movements of the limb and patient without risk of moving the fracture site, if a proper extension and immobilizing splint is used. Suspension is important when treating compound fractures and war wounds, for it facilitates dressing, irrigation and X-ray examination of the wound, besides allowing free movements of the limbs and of the patient. During treatment, the limb should be maintained in a slightly flexed position so as to avoid muscle strain and subsequent paralysis.

The damage done to the soft parts must not be overlooked; massage and care of the skin and circulation are important even in simple fractures, and are essential in war wounds.

In the treatment of fractures, the best appliance is that which permits the application of the above described principles. After study of the splints in use, the conclusion invariably arrived at is that the wire cradle type embodies most of the essential principles of treatment, and that the best splint of this type is the Hodgen wire cradle splint.

The advantages of the Hodgen splint can be summarized as follows:

(1) Immobility of the fracture site is ensured.

(2) The required degree of extension with gravity counter-extension is given, with easy control of extension, and counter extension neither painful nor injurious to the perineum and soft parts.

(3) Abduction and adduction are maintained.

(4) Extension is within the splint and leaves the limb mobile and free.

(5) Physiologic flexion is secured and muscle strain prevented.

(6) Mobility of the patient is ensured.

(7) The splint permits the treatment of injured soft parts.

(8) It can be modified for treatment of any fracture from pelvis to foot.

(9) A modification has been made for transport service.

